# Identification of sensory hair-cell transcripts by thiouracil-tagging in zebrafish

**DOI:** 10.1186/s12864-015-2072-5

**Published:** 2015-10-23

**Authors:** Timothy Erickson, Teresa Nicolson

**Affiliations:** Oregon Hearing Research Center and Vollum Institute, Oregon Health & Science University, 3181 SW Sam Jackson Park Road, Portland, OR 97239 USA

**Keywords:** TU-tagging, Zebrafish, Hair cell, Sensory hair cells, Transcriptomics, Gene expression, Transcriptional profiling, UPRT, Inner ear, Lateral line, Neuromast

## Abstract

**Background:**

Sensory hair cells are exquisitely sensitive to mechanical stimuli and as such, are prone to damage and apoptosis during dissections or *in vitro* manipulations. Thiouracil (TU)-tagging is a noninvasive method to label cell type-specific transcripts in an intact organism, thereby meeting the challenge of how to analyze gene expression in hair cells without the need to sort cells. We adapted TU-tagging to zebrafish to identify novel transcripts expressed in the sensory hair cells of the developing acoustico-lateralis organs.

**Methods:**

We created a transgenic line of zebrafish expressing the *T.gondii* uracil phospho-ribosyltransferase (UPRT) enzyme specifically in the hair cells of the inner ear and lateral line organ. RNA was labeled by exposing 3 days post-fertilization (dpf) UPRT transgenic larvae to 2.5 mM 4-thiouracil (4TU) for 15 hours. Following total RNA isolation, poly(A) mRNA enrichment, and purification of TU-tagged RNA, deep sequencing was performed on the input and TU-tagged RNA samples.

**Results:**

Analysis of the RNA sequencing data revealed the expression of 28 transcripts that were significantly enriched (adjusted *p*-value < 0.05) in the UPRT TU-tagged RNA relative to the input sample. Of the 25 TU-tagged transcripts with mammalian homologs, the expression of 18 had not been previously demonstrated in zebrafish hair cells. The hair cell-restricted expression for 17 of these transcripts was confirmed by whole mount mRNA *in situ* hybridization in 3 dpf larvae.

**Conclusions:**

The hair cell-restricted pattern of expression of these genes offers insight into the biology of this receptor cell type and may serve as useful markers to study the development and function of sensory hair cells. In addition, our study demonstrates the utility of TU-tagging to study nascent transcripts in specific cell types that are relatively rare in the context of the whole zebrafish larvae.

**Electronic supplementary material:**

The online version of this article (doi:10.1186/s12864-015-2072-5) contains supplementary material, which is available to authorized users.

## Background

Sensory hair cells are the highly specialized mechanoreceptors of the auditory, vestibular, and lateral line (acoustico-lateralis) organs in vertebrates. Due to their mechanical sensitivity, relative scarcity, and the complex anatomy of the acoustico-lateralis organs, hair cells have been a difficult cell type to dissociate, purify and analyze. Uncovering new hair cell-enriched transcripts would complement the genetic approaches that have identified many, but not all, of the genes required for the function of hair cells. The zebrafish is an excellent genetic model for hearing and balance [1, 2], however, only one study has attempted to experimentally define the zebrafish hair-cell transcriptome using dissociated macular cells from adults [3]. As such, larval zebrafish hair-cell gene expression remains poorly characterized. Uncovering additional hair cell-specific transcripts would both increase the usefulness of zebrafish as a model for hearing and balance disorders, and deepen our understanding of the development and function of vertebrate hair cells.

Previous transcriptional profiling studies of hair cells have used either manual cell sorting or fluorescence-activated cell sorting (FACS) to enrich for mammalian auditory and vestibular hair cells, as well as regenerating hair cells in the zebrafish lateral-line organ [3–9]. Although successful at enriching for rare cell types, the drawback of invasive cell-purification techniques is that they require extensive tissue manipulation that may disrupt endogenous patterns of gene expression. Furthermore, cell sorting captures the entire transcriptome of a cell, and does not discriminate between preexisting RNA and newly synthesized transcripts. For these two reasons, FACS may not be the best choice for analyzing dynamic changes in gene expression. To enrich for cell-type specific RNA from an intact organism, a number of innovative techniques have been developed, including INTACT nuclei purification [10–12], translating ribosome affinity purification (TRAP) [13–15], and thiouracil (TU) RNA tagging [16, 17]. To further our understanding of the biology of zebrafish hair cells, and to develop an alternative to invasive cell-purification techniques, in this study we describe the use of TU-tagging in zebrafish to label hair cell-expressed transcripts *in vivo*.

TU-tagging is a method to enrich for actively transcribed RNA from a specific cell type of interest. This is achieved through the cell type-restricted expression of the *Toxoplasma gondii* uracil phospho-ribosyltransferase (UPRT) enzyme together with the global application of its substrate, 4-thiouracil (4TU). UPRT-positive cells will preferentially convert 4TU to 4-thiouridine monophosphate, a thiol-substituted form of uridine that can be readily incorporated into nascent RNA. By taking advantage of the fact that thiol (sulfur-containing) groups do not normally exist in ribonucleic acids, thiol-tagged RNA from rare or difficult to isolate cell types (such as hair cells) can be biotinylated *in vitro* and selectively purified from the greater RNA pool [16, 17]. Moreover, because RNA is labeled in the live, intact organism, TU-tagging alleviates concerns about disrupting endogenous patterns of gene expression by invasive cell isolation techniques. We have created a transgenic line of fish that expresses an HA-epitope tagged UPRT enzyme and a red fluorescent protein (*Tg(myo6b:HA-UPRT-P2A-mCherry*)) under the control of the *myosin 6b* minimal promoter to restrict UPRT expression to the zebrafish auditory, vestibular and lateral-line hair cells. TU-tagged and input RNA samples were subjected to RNA sequencing and transcript abundance was analyzed by *DESeq* to identify putative hair cell-expressed transcripts. In all, we found 28 significantly enriched transcripts (adjusted *p*-value < 0.05), only seven of which were known to be expressed in zebrafish hair cells. Using whole mount mRNA *in situ* hybridization, we confirmed the hair cell-restricted expression of an additional 17 genes whose spatial expression pattern had not been previously described in zebrafish. To our knowledge, this is the first demonstration of TU-tagging in zebrafish, and suggests that this technique may be useful in other zebrafish cell types.

## Results

### Generation and characterization of *Tg(myo6b:HA-UPRT-P2A-mCherry)* transgenic fish

**Fig. 1:**
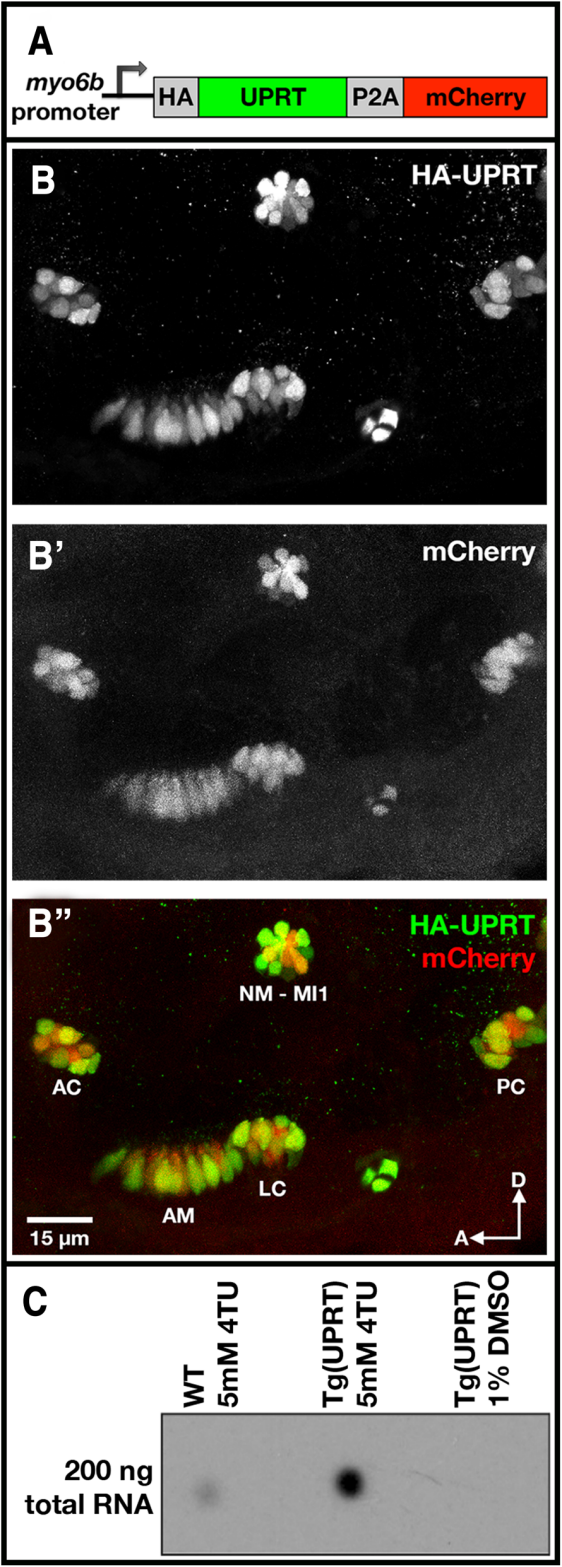
UPRT expression and activity in zebrafish hair cells. **a** Diagram of the construct used for Tol2 Gateway-mediated transgenesis. A minimal *myo6b* promoter was used to drive expression of the *HA-UPRT-P2A-mCherry* transgene in auditory, vestibular, and lateral line hair cells. **b-b**” Maximum projection images of immunolabeled HA-UPRT and fixed mCherry fluoresecence in inner ear hair cells of a 3 dpf *Tg(myo6b:UPRT)* larva. AC, anterior crista; AM, anterior macula; LC, lateral crista; PC, posterior crista. The focal plane includes a neuromast (NM-MI1). **c** Dot blot for TU-tagged, biotinylated total RNA demonstrating the enzymatic activity of UPRT in the hair cells of 5 dpf zebrafish larvae. Nontransgenic wild-type (WT) larvae exhibited low levels of 4TU incorporation in contrast to *Tg(myo6b:UPRT)* larvae when exposed to 5 mM 4TU for 3 h. RNA from *Tg(myo6b:UPRT)* larvae exposed to DMSO only did not exhibit any detectable biotinylation

Using the Tol2/Gateway system [18] we created transgenic zebrafish that expressed an HA-epitope tagged version of the *Toxoplasma gondii* UPRT enzyme in auditory, vestibular, and lateral line hair cells under control of the *myosin 6b* promoter (Fig. 1a). Additionally, we used a P2A-mCherry marker to visually score for transgenesis. We selected a line of *Tg(myo6b:HA-UPRT-P2A-mCherry)* (hereafter: *Tg(myo6b:UPRT)*) fish that exhibited bright, hair cell-restricted mCherry fluorescence, and confirmed UPRT expression by co-staining for the HA tag at 3 dpf (Fig. 1b).

Functionality of the *T.gondii* UPRT enzyme has not been previously demonstrated in zebrafish. To test if UPRT activity in zebrafish hair cells enhanced 4-thiouracil incorporation into nascent RNA, we treated 5  dpf wild type and *Tg(myo6b:UPRT)* larvae with either 1 % DMSO or 5 mM 4TU/1 % DMSO for 3 h. Total RNA was isolated, biotinylated *in vitro*, and dotted onto a membrane. TU-tagged, biotinylated RNA was detected with streptavidin-HRP (Fig. 1c). Wild type larvae exposed to 4TU did show some UPRT-independent labeling. However, the level of 4TU incorporation was greatly enhanced in *Tg(myo6b:UPRT)* larvae. RNA from *Tg(myo6b:UPRT)* larvae exposed to DMSO alone did not exhibit any detectable biotinylation. These dot blot results indicate that UPRT is functional when expressed in zebrafish hair cells.

### TU-tagging enriches for hair cell-expressed transcripts

**Fig. 2:**
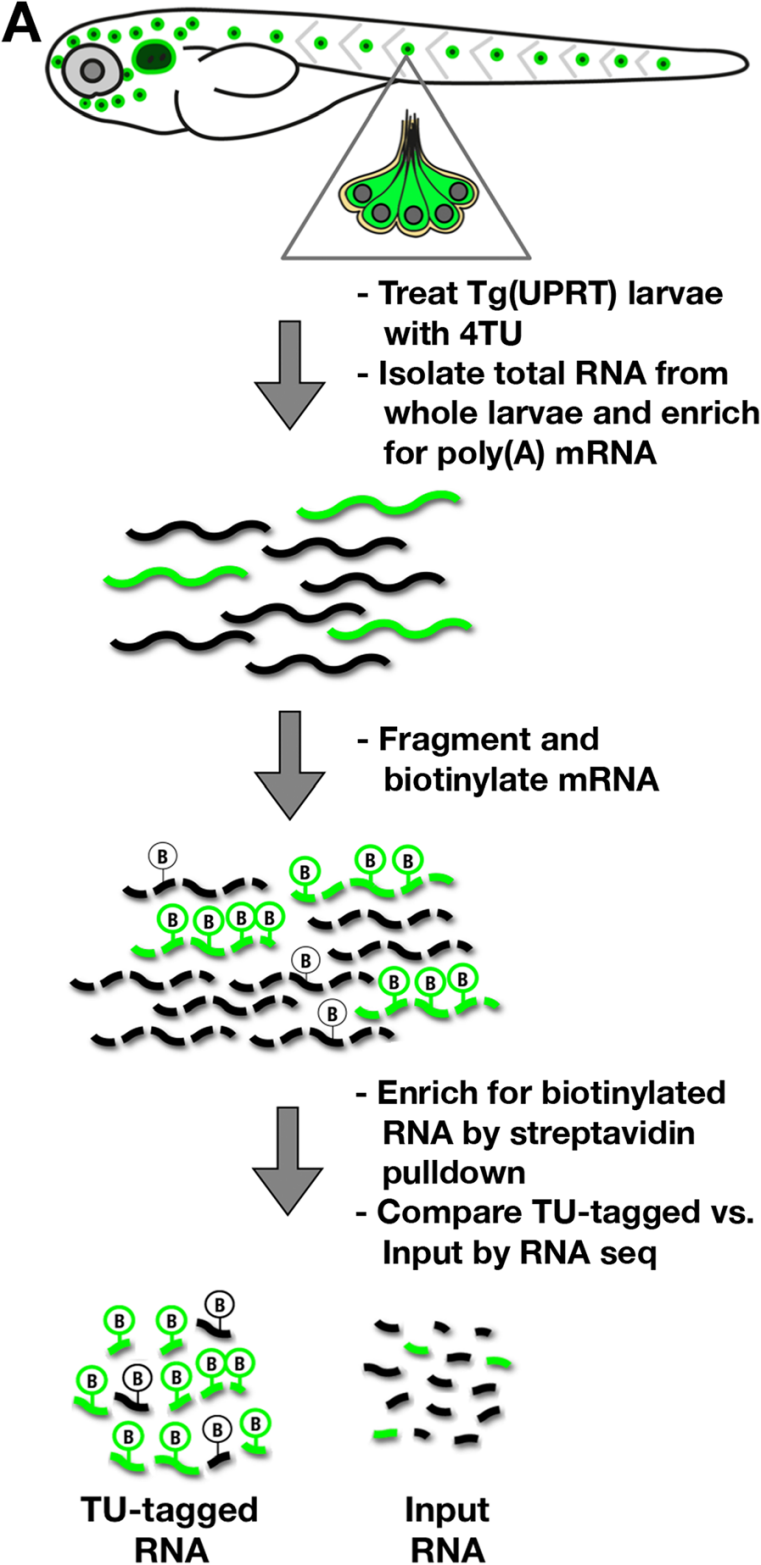
TU-tagging workflow diagram. Larvae (3 dpf) were exposed to 2.5 mM 4TU for 15 h and then homogenized to isolate total RNA. Purified Poly (**a**) mRNA was then fragmented and biotinylated for strepavidin-mediated pull down. RNAseq libraries were constructed and sequenced for comparison of transcript abundance between TU-tagged and input control RNA

To label and purify hair cell mRNA from zebrafish, we adapted the general strategy outlined in Gay et al. (see Methods and Fig. 2). We treated 3 dpf wild type and *Tg(myo6b:UPRT)* larvae with 2.5 mM 4TU/1 % DMSO for 15 h at 29 °C. Following total RNA extraction and poly(A) mRNA enrichment, the mRNA was fragmented, biotinylated, and TU-tagged fragments were isolated using streptavidin-mediated pulldown. Barcoded *Illumina* RNA seq libraries were prepared from the following four sources and sequenced on one lane of a HiSeq 2000 sequencer: [1] *Tg(myo6b:UPRT)* input (pre-pull down) RNA, [2] *Tg(myo6b:UPRT)* TU-tagged (pull down) RNA, [3] wild-type (non-transgenic) input RNA, and [4] wild-type TU-tagged RNA. For each of the experimental groups, we mapped the sequencing reads to the Zv9 zebrafish genome using *Tophat2* [19] and counted the number of reads aligning with each annotated gene region using *SeqMonk* [20]. Read counts were imported to *DESeq* [21] to determine statistically significant differences in transcript abundance between the input and TU-tagged samples derived from both *Tg(myo6b:UPRT)* and wild-type control larvae.

**Fig. 3:**
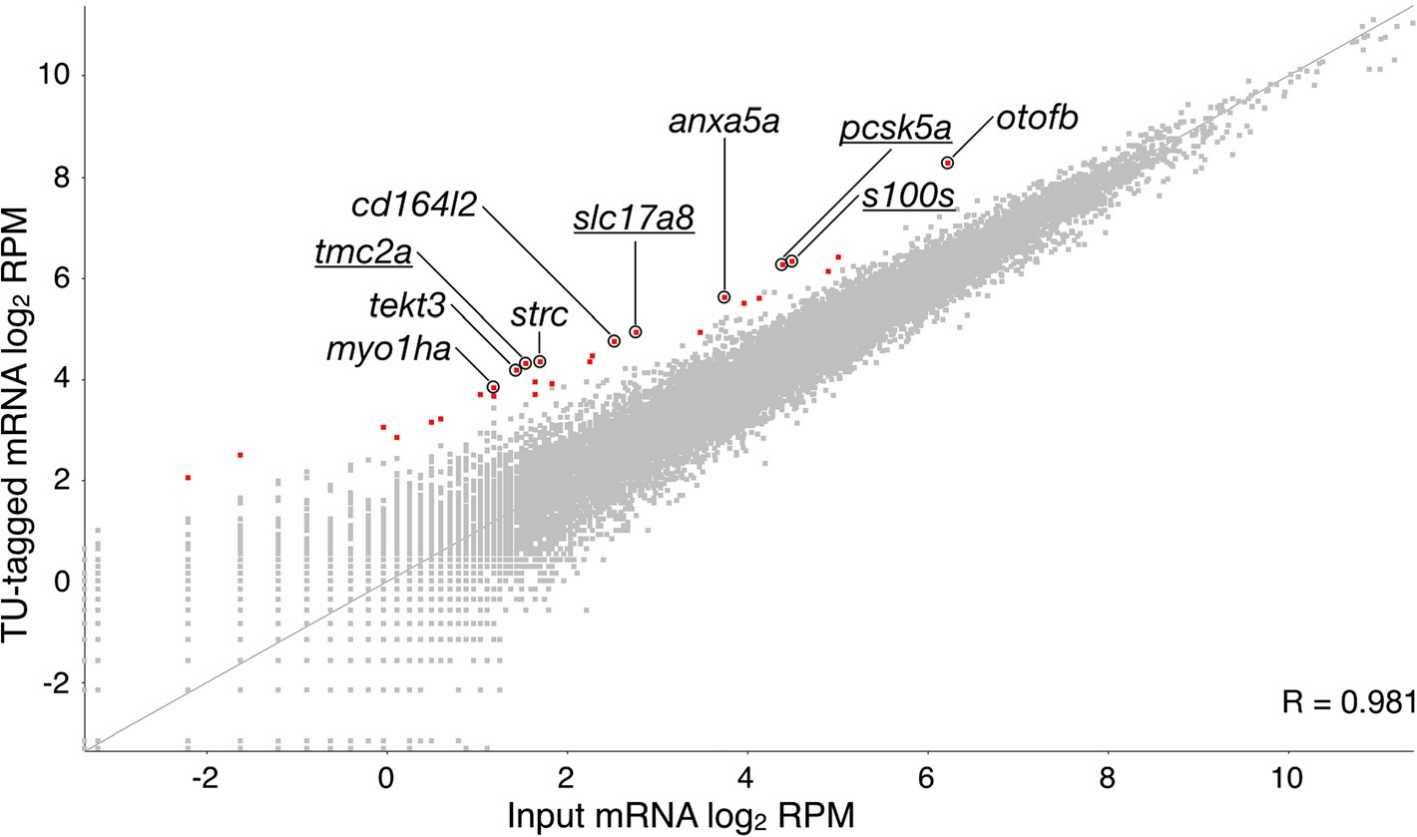
Enriched nascent transcripts in larval hair cells. SeqMonk scatter plot showing the correlation between the log_2_-transformed reads-per-million mapped reads (RPM) values for each zebrafish gene from the TU-tagged and input mRNA samples. The 28 significantly enriched TU-tagged genes (*DESeq* adjusted *p*-value < 0.05; Table 1) are indicated by the *red points* to the upper left of the diagonal. The top ten significantly enriched genes are annotated by name, with previously known zebrafish hair cell transcripts underlined

Our statistical analysis revealed 32 transcripts that were significantly enriched (adjusted *p*-value < 0.05) greater than 2-fold in the *Tg(myo6b:UPRT)* TU-tagged sample relative to the input (Additional file 1: Table S1). We filtered this list further by excluding four transcripts (*si:dkey-22f5.9*, *slc10a2*, *slc20a1a*, and *tmem27*) that were enriched >2-fold in the wild-type TU-tagged sample relative to the corresponding wild-type input (Additional file 2: Table S2), as the enrichment of these transcripts in non *Tg(myo6b:UPRT)* larvae was not related to hair cell-specific expression. As a result, we found 28 transcripts whose abundance was significantly enriched in the TU-tagged RNA sample (Fig. 3, Table 1).

**Table 1:** Significantly enriched TU-tagged genes

Gene name	Ensembl ID	Fold Enrich.	P-adj	Mouse Homolog (SHIELD FDR)	Zfish expression
*otof b*	ENSDARG00000020581	4.14	2.72E-09	*Otof* (5.58e-12)	Ear, NM
*tmc2a*	ENSDARG00000033104	7.29	1.96E-05	*Tmc2* (0.466)	Ear [30]
*pcsk5a*	ENSDARG00000067537	3.65	1.96E-05	*Pcsk5* (0.843)	Ear, NM [26]
*s100s*	ENSDARG00000036773	3.55	2.17E-05	*S100a1* (1.03e-6)	Ear, NM [27, 28]
*cd164l2*	ENSDARG00000096327	5.03	3.99E-05	*Cd164l2* (1.51e-11)	Ear, NM
*slc17a8*	ENSDARG00000057728	4.64	5.21E-05	*Slc17a8* (9.06e-7)	Ear, NM [29]
*STRC*	ENSDARG00000078845	6.36	6.90E-05	*Strc* (2.58e-10)	Ear
*tekt3*	ENSDARG00000045038	6.72	1.19E-04	*Tekt3* (0.035)	Ear, NM
*anxa5a*	ENSDARG00000026406	3.65	1.60E-04	*Anxa5* (0.102)	Ear, NM
*myo1ha*	ENSDARG00000061968	6.33	1.14E-03	*Myo1h* (na)	Ear, NM
*cabp2b*	ENSDARG00000052277	4.42	2.58E-03	*Cabp2* (5.96e-16)	Ear, NM [23]
*CR293520.1*	ENSDARG00000088059	6.29	3.27E-03	*Strc* (2.58e-10)	NM
*baiap2l2*	ENSDARG00000060933	5.90	3.27E-03	*Baiap2l2* (0.099)	Ear, NM
*myo15ab*	ENSDARG00000078474	4.36	3.72E-03	*Myo15* (6.65e-6)	Ear, NM
*si:dkey-229d2.6*	ENSDARG00000095684	17.53	4.73E-03	No homolog	ND
*si:rp71-68n21.12*	ENSDARG00000077382	4.82	6.04E-03	*Hcn4* (0.488)	Ear, NM
*myo6b*	ENSDARG00000042141	2.57	6.04E-03	*Myo6* (5.68E-04)	Ear, NM [24, 25]
*si:dkeyp-110e4.11*	ENSDARG00000071585	4.27	6.70E-03	*Stard10* (1.08e-5)	Ear, NM
*si:dkey-229d2.7*	ENSDARG00000092460	21.65	8.55E-03	*Kif5b* (0.356)	Ear
*GPR113 (2 of 2)*	ENSDARG00000093008	2.89	8.76E-03	*Gpr113* (0.771)	Taste buds
*CD37*	ENSDARG00000075515	7.63	1.13E-02	*Cd37* (0.912)	Ear, NM
*CR391998.1*	ENSDARG00000091817	2.76	1.17E-02	No homolog	ND
*GPX2*	ENSDARG00000089149	6.58	1.30E-02	*Gpx2* (2.15e-10)	Ear
*dnajc5b*	ENSDARG00000058147	6.11	2.07E-02	*Dnajc5b* (7.73e-10)	Ear, NM
*FAM188B2*	ENSDARG00000069867	7.11	2.25E-02	*Fam188b2* (na)	Ear, NM
*CABZ01086597.1*	ENSDARG00000088304	2.88	2.25E-02	No homolog	ND
*chrna9*	ENSDARG00000054680	4.23	4.05E-02	*Chrna9* (2.73e-15)	Ear, NM
*s100t*	ENSDARG00000055589	2.33	4.05E-02	*S100a1* (1.03e-6)	NM [27, 28]

DEseq analysis of the TU-tagged and input RNA seq read count values revealed 28 candidate hair cell-expressed genes. For each gene, the level of enrichment relative to the input and the adjusted *p*-value (P-adj) are indicated. Mouse homologs were identified by Ensembl database queries or BlastP similarity. For each mouse gene, the False Discovery Rate (FDR) from the SHIELD database is provided, low values indicating enriched expression in mouse hair cells. Tissue expression of the zebrafish genes is summarized in the last column. Expression patterns are described in this paper unless otherwise noted. Abbreviations: *NM* neuromast, *ND* not done

To determine if these *Tg(myo6b:UPRT)*-enriched transcripts were selectively expressed in zebrafish hair cells, we searched the *PubMed* and *ZFIN* [22] databases for data on spatial patterns of gene expression. Of the 28 enriched genes, only seven - *cabp2b*, *myo6b*, *pcsk5a*, *s100s*, *s100t*, *slc17a8*, and *tmc2a* - have been previously shown by *in situ* hybridization to be expressed in zebrafish sensory hair cells [23–30], while there was no data available for the remaining 21 (Table 1). For 18 of these 21 putative hair cell-enriched transcripts, we identified a homologous mouse gene by either querying the *Ensembl* database, or by *BLASTP* similarity. We used this homology information to search the Shared Harvard Inner-ear Laboratory Database (SHIELD), a repository for an RNA sequencing dataset derived from FAC-sorted mouse hair cells [4, 9]. We found that 16 of the 18 mouse homologs had detectable expression in either vestibular or auditory hair cells, and that 12 homologs were significantly enriched (FDR ≤0.1) in GFP+ hair cells relative to GFP- inner ear cells. Additionally, because the mouse Gene Ontology annotation is more detailed than that for zebrafish, we used the 23 unique identifiable mouse homologs of the entire *Tg(myo6b:UPRT)*-enriched zebrafish gene set to perform a Gene Ontology (GO) term analysis [31, 32]. Amongst the *Tg(myo6b:UPRT)*-enriched dataset, the significantly over-represented Biological Process GO terms are all related to hair-cell development and function (Corrected *p*-value < 0.01; Table 2). Taken together, these *in silico* analyses suggest that our TU-tagging experiment successfully enriched for hair cell-expressed genes in zebrafish.

**Table 2:** Biological process Gene Ontology (GO) term analysis of the TU-tagged gene set

GO ID	TERM	*P*-VALUE (adj)	ANNOTATED GENES
GO:0007605	sensory perception of sound	1.79E-08	*Myo6*, *Myo15*, *Strc*, *Slc17a8*, *Tmc2*, *Chrna9*, *Otof*
GO:0050954	sensory perception of mechanical stimulus	4.31E-08	*Myo6*, *Myo15*, *Strc*, *Slc17a8*, *Tmc2*, *Chrna9*, *Otof*
GO:0050910	detection of mechanical stimulus involved in sensory perception of sound	1.25E-04	*Strc*, *Tmc2*, *Chrna9*
GO:0042472	inner ear morphogenesis	6.88E-04	*Myo6*, *Myo15*, *Strc*, *Chrna9*
GO:0050974	detection of mechanical stimulus involved in sensory perception	9.92E-04	*Strc*, *Tmc2*, *Chrna9*
GO:0042471	ear morphogenesis	1.38E-03	*Myo6*, *Myo15*, *Strc*, *Chrna9*
GO:0050982	detection of mechanical stimulus	3.32E-03	*Strc*, *Tmc2*, *Chrna9*
GO:0006811	ion transport	5.10E-03	*Myo6*, *S100a1*, *Tmc2*, *Kif5b*, *Stard10*, *Slc17a8*, *Hcn4*, *Chrna9*
GO:0048839	inner ear development	6.46E-03	*Myo6*, *Myo15*, *Strc*, *Chrna9*

Significantly enriched (adjusted *p*-value < 0.01) Biological process GO terms associated with the mouse homologs of the zebrafish TU-enriched genes

### Using whole mount *in situ* hybridization to characterize the spatial expression of TU-tagged transcripts in zebrafish

**Fig. 4:**
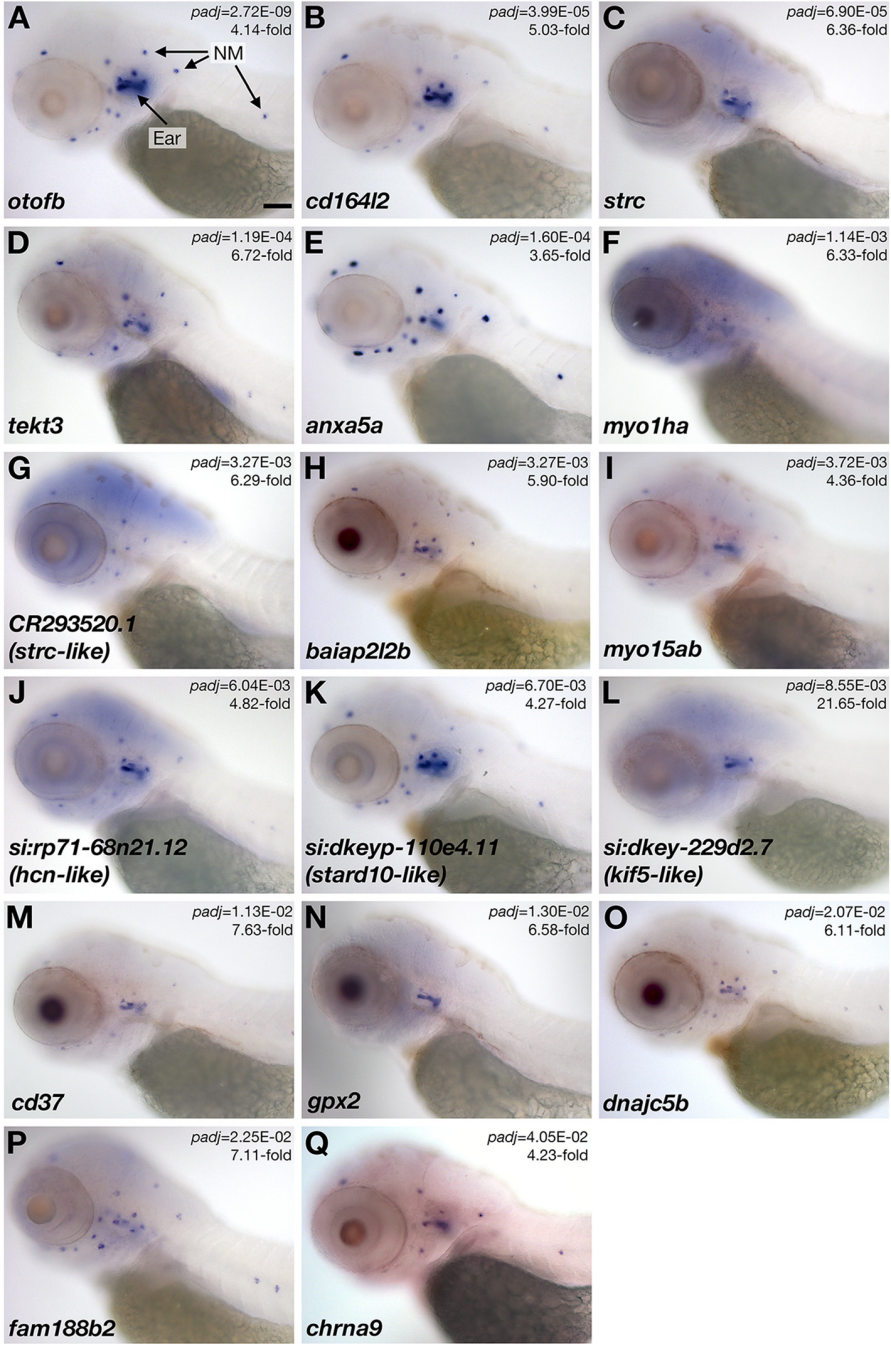
Validation of hair-cell specific gene expression by whole mount mRNA *in situ* hybridization. Panels **a**-**q** show the mRNA *in situ* hybridization patterns for 17 of the uncharacterized TU-enriched genes. Lateral views (dorsal up; anterior to the left) of the head and anterior trunk of 3 dpf larvae are depicted. In each panel, the focal plane includes sensory epithelia of the inner ear and neuromasts (NM), as indicated in panel **a. ** The *DESeq* adjusted *p*-value (*padj*) and fold-enrichment of the transcript in the TU-tagged mRNA sample are indicated for each gene. Scale bar in A = 100 μm

Of the 28 significantly enriched TU-tagged transcripts, the spatial expression pattern of 21 genes has not been reported in zebrafish. To directly test if these genes are expressed in zebrafish hair cells at 3 dpf, we performed *in situ* hybridization (ISH) for those 18 genes that had clearly identifiable mammalian homologs. In total, we were able to confirm hair cell-restricted expression for 17 of these 18 TU-enriched transcripts (Fig. 4). We found that one of the previously uncharacterized genes – a zebrafish ortholog of *gpr113* – was expressed in taste buds, and not in hair cells (data not shown). Control sense probes for *anxa5a*, *cd164l2*, *otofb*, *strc*, and *tekt3* did not yield specific signals in hair cells (data not shown). Considering both the previously reported expression patterns and the 17 new patterns described here, these TU-enriched genes were primarily if not exclusively expressed in hair cells. Most genes (*n* = 18) were detected in both ear and lateral-line hair cells, while four genes were primarily expressed in the ear (*gpx2*, *si:dkey-229d2.7*/*kif5-like, strc*, and *tmc2a*), and the expression of two genes were detected in the lateral line organ only (*CR293520.1*/*strc-like* and *s100t*). These ISH results confirm that our TU-tagging experiment successfully enriched for auditory, vestibular, and lateral line hair-cell transcripts.

## Discussion

Our results demonstrate that TU-tagging is a viable, noninvasive method for identification of cell type-specific mRNA in zebrafish. Specifically, we employed this profiling technique to identify genes that are selectively expressed during development in sensory hair cells. By adapting the method of TU labeling of transcripts to a larval stage (3 dpf) in zebrafish, we found 28 transcripts that were significantly enriched in the TU-tagged mRNA sample of newly developed hair cells compared to the untagged input mRNA at the same developmental stage. Using *in situ* hybridization, we confirmed the specific expression pattern of 17 genes in hair cells that have not been previously described in zebrafish. Our work has substantially added to the number of confirmed hair cell-enriched transcripts in zebrafish and serves as an example of how TU-tagging can be used for characterization of newly synthesized transcripts in a rare cell type.

Our TU-tagging experiment sought to purify transcripts from auditory, vestibular and lateral-line hair cells from whole larvae without any prior tissue enrichment. We estimate that hair cells represent <1 % of the total cell number in a 3 dpf zebrafish larva (~750 hair cells in a larva of > >100,000 cells [33]). In addition to being scarce, zebrafish hair cells are clustered at different locations within the otic vesicle or distributed in neuromasts at the surface of the skin, making the enrichment of hair cell transcripts a demanding test for TU-tagging. Ideally, any RNA-enrichment experiment would identify hair cell-expressed transcripts with high specificity and high sensitivity; that is, identify only hair cell transcripts and detect even the rarest ones, regardless of whether they were also expressed in other cell types. Given our experimental design, we found that TU-tagging enriched for hair cell transcripts with good specificity, but poor sensitivity. This means that the majority of our significantly enriched transcripts are *bona fide* hair cell-expressed genes. However, the experiment was not sensitive enough to identify anything other than hair cell-specific transcripts. The *in situ* hybridization experiments confirm the limitations on sensitivity; all of the significantly enriched TU-tagged transcripts were exclusive to hair cells. Our experiment did not identify known hair cell-expressed transcripts that are also robustly expressed in other tissues, such as the deafness genes *pcdh15a*, *cacnad1a*, or *cdh23* (Additional file 1: Table S1). While our TU-tagging experiment successfully identified novel hair cell-specific transcripts in zebrafish, as performed, it was not an effective tool for analyzing the entire hair-cell transcriptome.

To improve the sensitivity of TU-tagging in zebrafish, manual tissue enrichment prior to RNA isolation is an option. This approach, similar to that taken in mice by Gay et al. [17], is more cumbersome in zebrafish due to the large number of small larvae required for the experiment. An alternative is to use adult tissues if the developmental stage is not an issue. Other possible changes to the experimental protocol could include shortening the duration and concentration of 4TU exposure, as this may reduce UPRT-independent labeling in non-target cell types. Furthermore, performing the experiment with discrete biological replicates will increase the statistical power during data analysis and may increase the sensitivity of transcript detection. However, due to the UPRT-independent thiol-labeling we observed, it is likely that TU-tagging of rare cell types will always have a signal-to-noise problem to some extent.

## Conclusions

Despite the limitations on sensitivity, in our hands the TU-tagging method was robust using undissected larvae, revealing 17 hitherto unknown cell type-specific transcripts in developing zebrafish hair cells. In the context of the whole larva, acousticolateralis hair cells are a relatively rare cell type, thus this approach is likely to be useful for analyzing gene expression in other tissues or specific types of cells as well. The major appeal of TU-tagging is the ability to spatially control the expression of UPRT and temporally control the exposure to 4TU. The ability of TU-tagging to discriminate between newly synthesized and pre-existing transcripts will enhance future studies of changes in gene expression during dynamic processes such as development or synaptic plasticity.

## Methods

### Zebrafish husbandry

Zebrafish were cared for in accordance with standard protocols and overseen by the Institutional Animal Care and Use Committee at Oregon Health and Sciences University. Larvae were obtained from pair-wise natural matings and kept at 29 °C in E3 embryo media (5 mM NaCl, 0.17 KCl, 0.33 mM CaCl2 and 0.33 mM MgSO4).

### Generating UPRT transgenics

The Tol2 Gateway transgenesis vectors and *Tg(myo6b:HA-UPRT-P2A-mCherry)* transgenic fish were generated essentially as previously described (Kwan et al. [18]). To make the HA-epitope tagged UPRT middle entry vector, the uracil phosphoribosyltransferase (UPRT) gene from *Toxoplasma gondii* (a kind gift from the lab of Richard Goodman) was amplified by PCR using custom attB1F and attB2R Ultramer oligonucleotide primers (Integrated DNA Technologies) containing an in-frame HA-epitope tag sequence upstream of the UPRT-specific sequence on the forward primer. Similarly, the P2A-mCherry 3' entry vector was generated from the pME-NLSmCherry (#233) template using attB2F-attB3R oligonucleotide primers containing an in-frame P2A viral peptide sequence [34] upstream of the mCherry-specific sequence on the forward primer. The resulting PCR products were cloned into the pDONR-221 or pDONR-P2R-P3 vectors respectively by standard protocols and verified by sequencing. The final Tol2 *myo6b:HA-UPRT-P2A-mCherry* transgenesis construct was assembled with the pDestTol2pA2 (#394) and p5e*—6.5myo6b* minimal promoter [29] vectors using the standard LR cloning procedure and verified by a diagnostic restriction digest. A stable UPRT transgenic line was chosen based on bright, hair cell-specific mCherry fluorescence.

### TU-tagging: 4TU application and total RNA purification

4-thiouracil (Sigma – 440736) was dissolved in DMSO (Sigma – D8418) to a concentration of 0.5 M and aliquots stored at −20 °C. Progeny from outcrosses of *Tg(myo6b:HA-UPRT-P2A-mCherry)* (*Tg(myo6b:UPRT)* for short) transgenic adults were enzymatically dechorionated at 36 h post fertilization (hpf), sorted for mCherry fluorescence at 48 hpf, and raised in 0.2 μm filter-sterilized E3 embryo media until 3 days post fertilization (dpf). Using a 70 μm nylon Falcon cell strainer (Fisher Scientific), approximately 250 transgenic larvae (3.5 dpf) were transferred to Petri dishes (approximately 50 larvae per dish) containing 20 ml of 2.5 mM 4TU / 0.5 % DMSO in filter-sterilized E3 and incubated in 4TU for 15 h at 29 °C. Following the incubation, larvae were anesthetized by the addition of MESAB to the Petri dishes, concentrated in a 70 μm nylon Falcon cell strainer, and transferred to a 1.5 ml microfuge tube. After excess liquid was removed, larvae were immediately homogenized in 1.25 ml of TRIzol reagent (Ambion – 15596–026) and processed according to the manufacturer’s protocol. RNA quality was confirmed using the Agilent RNA Pico kit.

### RNA processing and purification of TU-tagged RNA

#### DNAse-treatment

Total RNA was treated with DNase (Ambion Turbo DNA-free – AM1907) as per the manufacturer’s protocol. DNAse-treated total RNA was precipitated in 1/10 volume of NaOAc, 200 ng/μL glycogen, and 2.5 volumes of ice-cold 100 % ethanol for 1 h at −80 °C. Precipitates were spun at maximum speed for 15 min at 4 °C, washed in ice-cold 75 % ethanol, and dissolved in RNase-free water.

### mRNA purification, fragmentation and biotinylation

Poly-adenylated mRNA was purified using the Ambion Dynabeads mRNA Purification Kit (Ambion – 61006) according to the manufacturer’s protocol. Purified mRNA was fragmented for 4 min at 94 °C using the NEBNext Magnesium RNA Fragmentation Module (NEB – E6150S) to approximately 200–500 bases, recovered by ethanol precipitation, and dissolved in 50 μl RNase-free water. TU-tagged RNA was biotinylated using EZ-Link HPDP-Biotin (Thermo Scientific – 21341) by the addition of 25 μl 4x TE and 25 μl 1 mg/ml EZ-Link (dissolved in DMF) to the 50 μl RNA. Following a 3 h incubation at room temperature in the dark, excess biotin was removed by a chloroform/isoamyl alcohol (24:1) extraction as described [35]. The RNA was recovered by ethanol precipitation, at which point 80 ng of RNA was set aside as the “input” RNA sample.

### RNA dot blot

To test if UPRT expression in hair cells increased the rate of 4TU incorporation into nascent RNA relative to non-transgenic larvae, 5 dpf wild type or *Tg(myo6b:UPRT)* larvae were treated with 5 mM 4TU/1 % DMSO for 1.5 h at 29 °C. Total RNA was extracted using TRIzol reagent and, omitting the DNAse, mRNA-enrichment, and fragmentation steps, 10 μg was biotinylated as described above. Following reaction clean up with the Qiagen RNeasy mini kit (Qiagen – 74104), 250 ng of total RNA was pipetted directly onto a PVDF membrane and immobilized using the “Auto Cross Link” setting on a Stratalinker 1800 UV Crosslinker. The membrane was blocked for 15 min in 1x PBS/1 mM EDTA/1 % SDS at room temperature on an orbital shaker. After incubating in a 1:5000 dilution of 1 mg/ml Streptavidin-HRP (Thermo Scientific – 21126) in block for 10 min, the membrane was washed 1 × 10 min in block, 3 × 5 min in 1x PBS/0.1 % SDS, and 1x5 min in 1x PBS. Chemiluminescent detection was done using Pierce SuperSignal West Pico Chemiluminescent Substrate (Thermo Scientific – 34077) and GeneMate Blue Ultra Autorad Film (BioExpress – F-9029-8x10).

### Purification of TU-tagged RNA

Biotinylated, TU-tagged RNA was purified using μMacs Streptavidin kit (Miltenyi Biotec – 130-074-101). The biotinylated RNA sample volume was adjusted to 50 μl with RNase-free water, heated to 65 °C for 2 min, and then placed on ice. The sample volume was adjusted to 100 μl with MPG buffer (100 mM Tris–HCl pH7.4, 10 mM EDTA pH8, 1 M NaCl, 0.02 % Tween-20) and incubated for 5 min at room temperature with 100 μl μMacs streptavidin beads. Beads were washed once with 100 μl of 65 °C MPG buffer, followed by 3 washes with 100 μl room temperature MPG. TU-tagged RNA was eluted from the beads with 2 × 100 μl 65 °C 100 mM DTT. The combined 200 μl sample was cleaned up and concentrated using the Qiagen RNeasy MinElute cleanup kit (Qiagen – 74204), thereby comprising the “TU-tagged” RNA sample.

### RNA sequencing

RNA seq libraries were constructed using the TruSeq RNA sample preparation kit v2 (Illumina RS-122-2001) according to the manufacturer’s protocol. “Input” and “TU-tagged” stranded libraries were sequenced on a single lane of an Illumina HiSeq 2000, producing 100 base pair single end reads. The sequencing depth for all four samples was similar.

### Bioinformatics

The RNA seq reads were processed by *Trimmomatic* v0.32 [36] to remove Illumina adaptor sequences and discard sequence reads shorter than 36 bases. Trimmed reads were mapped against the Zv9 version of the zebrafish genome using *Tophat* v2.0.12 using the “--b2-sensitive” option [19]. The resulting BAM file was imported into *SeqMonk* v0.29 [20] for data visualization and quantification (Scatter plot, see Fig. 3). For the purpose of counting the RNA seq reads assigned to each annotated gene region, probes were defined as “Gene” features, including 250 bases up- and downstream. Duplicated reads were discarded and counted only once. The resulting raw read count table was imported into *DESeq* v1.14.0 [21] for normalization and statistical analysis of differential transcript abundance between the “Input” and “TU-tagged” RNA samples. Prior to statistical analysis, the bottom 20 % of low-count genes was filtered from the data set [37]. Because all “Input” and “TU-tagged” samples were pooled, dispersion estimates were done using the options for data without discrete replicate sets (method = “blind”,sharingMode = “fit-only”,fitType = “local”). Genes whose transcripts were significantly enriched (adjusted *p*-value < 0.05) in the “TU-tagged” RNA sample were considered for further analysis.

### Immunostaining and in situ hybridization

Larvae were fixed in 4 % paraformaldehyde/PBS for 4 h at room temperature followed by 5 × 5 min washes in 1x Phosphate Buffered Saline/0.1 % Tween-20 (PBST). To permeabilize, fixed larvae were rinsed twice in water and, after removing all liquid, submerged in −20 °C acetone for 3 min. Larvae were rinsed twice in water, washed 3 × 5 min in 1xPBS/0.01 % Tween-20, and blocked in FSGGB (0.5 % fish skin gelatin, 1 % goat serum, 1 % BSA, 1x PBS, 0.02 % sodium azide) at room temperature for 2 h on a Nutator mixer. To label HA-tagged UPRT, 3 dpf wild type and *Tg(myo6b:UPRT)* larvae were incubated in a 1:750 dilution of rat anti-HA clone 3F10 antibody (Roche – 1186742300) in FSGGB overnight at 4 °C, washed 5 × 15 min in 1x PBS/0.01 % Tween-20, incubated in a 1:1000 dilution of Alexa Fluor 488 goat anti-rat IgG (Life Technologies – A-11006), and washed again 5 × 15 min in 1x PBS/0.01 % Tween-20. Specimens were mounted on a depression slide in 1.2 % low-melting point agarose and imaged on a Zeiss LSM 700 confocal microscope using Zeiss *Zen* acquisition software.

Whole mount mRNA *in situ* hybridization (ISH) and probe synthesis was performed essentially as described [38, 39]. Using the SuperScript® III One-Step RT-PCR kit (Life Technologies – 12574–018), probe templates were amplified from 3 to 5 dpf total zebrafish RNA using gene-specific oligos with T3 and T7 RNA polymerase sites on the 5'-end of forward and reverse primers, respectively. Specimens were mounted on a depression slide in 1.2 % low-melting point agarose and imaged on a Leica DMLB microscope fitted with a Zeiss AxioCam MRc 5 camera using Zeiss *AxioVision* acquisition software (Version 4.5).

## Additional files

Additional file 1: Table S1. TU-tagged versus input RNAseq data from *Tg(myo6b:UPRT)* transgenic larvae. (XLSX 2308 kb) Additional file 2: Table S1. TU-tagged versus input RNAseq data from wild-type control larvae. (XLSX 2337 kb)
